# The pleasures of sad music: a systematic review

**DOI:** 10.3389/fnhum.2015.00404

**Published:** 2015-07-24

**Authors:** Matthew E. Sachs, Antonio Damasio, Assal Habibi

**Affiliations:** Brain and Creativity Institute, Dornsife College of Letters Arts and Sciences, University of Southern CaliforniaLos Angeles, CA, USA

**Keywords:** sad, music, neuroimaging, music therapy, depression

## Abstract

Sadness is generally seen as a negative emotion, a response to distressing and adverse situations. In an aesthetic context, however, sadness is often associated with some degree of pleasure, as suggested by the ubiquity and popularity, throughout history, of music, plays, films and paintings with a sad content. Here, we focus on the fact that music regarded as sad is often experienced as pleasurable. Compared to other art forms, music has an exceptional ability to evoke a wide-range of feelings and is especially beguiling when it deals with grief and sorrow. Why is it, then, that while human survival depends on preventing painful experiences, mental pain often turns out to be explicitly sought through music? In this article we consider why and how sad music can become pleasurable. We offer a framework to account for how listening to sad music can lead to positive feelings, contending that this effect hinges on correcting an ongoing homeostatic imbalance. Sadness evoked by music is found pleasurable: (1) when it is perceived as non-threatening; (2) when it is aesthetically pleasing; and (3) when it produces psychological benefits such as mood regulation, and empathic feelings, caused, for example, by recollection of and reflection on past events. We also review neuroimaging studies related to music and emotion and focus on those that deal with sadness. Further exploration of the neural mechanisms through which stimuli that usually produce sadness can induce a positive affective state could help the development of effective therapies for disorders such as depression, in which the ability to experience pleasure is attenuated.

## Introduction

Humans have long devoted effort and attention to the making and consuming of art that portrays and conveys misery. The ancient Greeks were known for staging tragedies that were widely popular; to this day, films and novels that deal with heartache and despair become bestsellers and garner critical attention. The phenomenon is seen across cultures and art forms. Classical music exhibits the phenomenon abundantly. Folk music, such as the Portuguese Fado (Nielsen et al., 2009) or the Irish Lament (O’Neill, 1910), often expresses sadness and grief. Sad-sounding motifs even permeate many modern-day American pop songs (Schellenberg and von Scheve, 2012).

Sadness in everyday life, however, is hardly pleasant. It is one of the six basic emotions (along with fear, happiness, anger, surprise, and disgust) and it results in feelings that most humans prefer not to experience. As is the case with other negative emotions, the importance of sadness throughout human history and across cultures can be explained through the evolutionary advantage that it confers (Ekman, 1992). Sadness results from a perceived loss, such as the loss of a valued object, the loss of health, the loss of status or of a relationship, or the loss of a loved one. It is a complex bodily and neural state, resulting in feelings of low energy, social withdrawal, low self-worth, and a sense of limited horizon of the future (Harter and Jackson, 1993; Damasio, 1999; Mee et al., 2006; Hervas and Vazquez, 2011).

Sad music can be defined objectively, based on its acoustical properties, and subjectively, based on a listener’s interpretation of the emotion that the composer is assumed to have conveyed. The musical features generally associated with “sadness” include lower overall pitch, narrow pitch range, slower tempo, use of the minor mode, dull and dark timbres, softer and lower sound levels, legato articulation, and less energetic execution (Juslin and Laukka, 2004). The emotional content of music can also be described on a bi-directional space of valence and arousal. In this view, sad music is defined as music with low valence and low arousal (Trost et al., 2012). Others classify music as sad based on either the emotion that is perceived or the emotion that is induced. This is usually determined by directly asking participants which emotion they believe is being expressed by the music or which emotion they feel when listening to the music (Guhn et al., 2007). The lyrics of popular songs and the poetry of classical pieces can play an important role in defining music as sad as they can trigger memories that the listener associates with sadness (Van den Tol and Edwards, 2013), such as themes of regret and lost love (Mori and Iwanaga, 2013).

Given that in most circumstances sadness is unpleasant, how then can it be associated with pleasure when expressed through music? Herein lies the so-called “tragedy paradox”, the seemingly contradictory idea that humans work to minimize sadness in their lives, yet find it pleasurable in an aesthetic context. The Athenian philosophers of the Pre-Christian era were the first to discuss this matter formally, proposing that art pertaining to negative emotions provides rewards that other art cannot provide. Aristotle, for example, spoke of how tragic theater allowed the audience to experience rapidly, and subsequently purge itself, of negative emotions, a beneficial outcome known as catharsis (Schaper, 1968). Philosophers and psychologists continue to explain the human attraction to sad art in terms of the psychological rewards that are associated with it.

There is room for disagreement, however, regarding the exact relationship between sad music and the associated pleasurable response. Many believe that music perceived as sad does not produce feelings of sadness and instead directly produces a positive affective state (Kivy, 1991). Others argue that, as is the case with *Schadenfreude*, pleasurable sadness can be viewed as a “mixed” emotion in which positive and negative affects are experienced simultaneously (Juslin, 2013). A third position is that sad music does induce feelings of sadness and that this negative affect is then made positive (Vuoskoski et al., 2011).

The recent emergence of new tools in cognitive science and neuroscience provides the possibility of investigating the relationship between perceived sadness in music and positive affect. By investigating how the brain responds to music listening, aesthetic judgment, and emotional processing, it is possible to gain a better understanding of how and why certain auditory stimuli eventually culminate in a pleasurable response.

In this article, we attempt to bring together findings from philosophy, psychology, and neuroscience in order to arrive at a framework for how sad music becomes pleasurable. We also propose ways of assessing the validity of the framework using neuroimaging and suggest how the available facts may be applicable to mental health interventions.

## The Tragedy Paradox: Philosophical and Psychological Approaches

The earliest attempts to reconcile the “tragedy paradox” came from philosophy and can be broadly organized into two main schools of thought. The “cognitivists” argue that music does not evoke real emotions, but that emotion can nonetheless be perceived in the structure of music, which, in turn, evokes reminders of the feelings associated with that emotion (Kivy, 1991). Cognitivists posit that emotive moments in music occur much too quickly to result in a full-fledged feeling of that emotion and, therefore, music can only act as a tour guide of past emotions (Hindemith, 1961).

On the other hand, the “emotivists” claim that music does induce real emotions in the listener (Levinson, 1990). Within the emotivist school of thought, however, there is still disagreement over the exact nature of the inducible emotions. Some emotivists argue that the emotional response is of a different sort than the kind experienced in everyday life. “Music-sadness” cannot be the same as “life-sadness”, they contend, because the environmental conditions necessary for that emotion are not present (Hospers, 1969). Given the inherently unpleasant nature of sadness, the pure fact that music expressing negative valence can even be found pleasant is proof enough that listeners do not feel sad. Instead, one is left only with responses such as awe, transcendence, and chills, which are inherently pleasurable, but do not entail or require the clear goal-oriented action that basic emotions promote (Scherer, 2004; Konečni, 2005).

Other “emotivists”, such as the philosopher Jerrold Levinson, argue that sad music does induce genuine sadness, and that this response is inherently rewarding. In his account, Levinson lists eight different benefits that can arise from the feeling of sadness evoked by music with a negative valence: *catharsis*, the purging of negative emotions, *apprehending expression*, an improved understanding of the emotions expressed in a piece of art, *savoring feeling*, the satisfaction that arises from simply feeling any emotion in response to art, *understanding feeling*, the opportunity to learn about one’s feelings*, emotional assurance*, the confirmation in one’s ability to feel deeply, *emotional resolution*, the knowledge that an emotion state has been, and can be, regulated, *expressive potency*, the pleasure that arises from expressing one’s feelings, and *emotional communion*, a connection to the feelings of the composer or other listeners (Levinson, 1990).

More recently, large-scale surveys in which participants were asked to provide their motives for listening to sad music have revealed that people often cite similar benefits to the ones described by Levinson (Garrido and Schubert, 2011). Furthermore, when participants were specifically asked about each of Levinson’s eight rewards relative to their justification for listening to sad music over happy music, they were more likely to associate sad music with the rewards of understanding feelings, emotional assurance, savoring feelings, emotional communion, and emotional resolution (Taruffi and Koelsch, 2014). Additional justifications included the trigger of specific memories, the distraction from current problems (Van den Tol and Edwards, 2013), the engagement of imaginative processes, and the experience of intense emotions without real-life implications (Taruffi and Koelsch, 2014).

Levinson’s ideas, and the ensuing survey data, point to a central mechanism by which sad music can become enjoyable: by triggering a number of psychological processes that are pleasurable to begin with. However, neither can fully explain how the association between sad music and psychological rewards arises or why this association is more likely to occur with sad music than with happy music. Sad music may in fact arouse feelings of connectedness and these feelings may be inherently pleasurable, but the question of how and why sad music allows one to feel more connected to others remains.

### Proposed Psychological Theories

A different line of research attempts to elucidate the relationship between sad music and affective response by exploring the underlying cognitive processes. Based on the notion that positive emotions, such as joy, are often linked to pleasure, while negative emotions are often linked to displeasure, Schubert (1996) proposed that negative-valence music is perceived as sad, but that this perception of negativity does not produce displeasure because the stimuli are considered to be “aesthetic” and therefore not actually harmful. In the wake of the dampened displeasure provided by the aesthetic context, a pleasurable response arises from the experience of arousal that the music produces. This theory provides a testable model for how sad music can be linked to pleasure, yet it does not clarify why other negative-valence stimuli, such as fear-inducing music, are generally not enjoyed.

In an attempt to address this question, Huron (2011) suggested that the hormone prolactin is responsible for enabling the enjoyment of sad music. Prolactin is released by endocrine neurons in the hypothalamus in response to tears and to the experience of negative emotions such as grief, sadness, and, more generally, stress (Turner et al., 2002). In such situations, its release encourages attachment and pair bonding as suggested by the fact that levels of prolactin fluctuate when people become parents, hear their children cry, or are mourning a recently deceased spouse (Lane et al., 1987; Delahunty et al., 2007). Huron proposes that the release of prolactin serves to comfort and console, to counteract the mental pain at the root of the negative emotion. He states that music simulates real sadness, which tricks the brain into engaging a normal, compensatory response, i.e., the release of prolactin. But because the listener is aware of the fact that they are not actually in a stressful or grief-inducing situation, the consoling effect of the hormone is produced in the absence of the mental pain that normally precedes it. The fact that the enjoyment of sadness varies greatly from person to person can be explained by differences in personality, emotional reactivity, cultural norms, biology and learned associations (Huron, 2011). No study to date has yet tested levels of prolactin in participants listening to music that evokes other negative emotions and thus this idea remains untested.

Like Schubert’s, Huron’s theory does not clarify why music is unique in its ability to produce this comforting after-effect. According to his view, other sad stimuli that simulate mental pain should be found pleasurable as well, such as sad faces or sad affective words. But existing research has suggested that this is not the case as the subjective report of experienced pleasure decreased when participants were presented with a sad photo (Wild et al., 2001).

A third proposal comes from Juslin’s BRECVEMA model, which describes eight mechanisms by which music can induce emotions: *brain stem reflexes, rhythmic entrainment, evaluative conditioning, contagion, visual imagery, episodic memory, musical expectancy*, and *aesthetic judgment* (Juslin, 2013). These mechanisms can work independently and as a group. A mixed emotion, such as pleasurable sadness, can be understood as the result of two different mechanisms generating different affective responses simultaneously. A sad piece of music might evoke a negative affect through the emotional contagion mechanism, which involves feeling the emotions that are recognized in external stimuli, and might evoke a positive affect through the aesthetic judgment mechanism, which involves deciding that the piece of music is aesthetically pleasing. In this account, the sad affective response does not lead to a joyful response, but rather sad music itself produces both sorrow and joy simultaneously (Juslin, 2013).

### Do Listeners Actually Feel Sad?

One common thread that runs through the available theories is that music that expresses sadness is enjoyed when the perceiver recognizes that the stimulus is not an immediate threat but is aesthetic instead. The fundamental disagreement concerns whether or not people actually feel sad when listening to sad music that they regard as pleasurable.

When people are directly asked the question, the responses vary. Roughly 25% say that they experience genuine sadness and the rest report that they experience some other, albeit related, emotion, most often, nostalgia (Huron, 2011). However, self-reports made in the context of emotional experience may provide inaccurate results since the difference between emotional perception and emotional experience may not be clear or equal to everyone. In studies in which the researchers made a clear distinction between “perceived” and “felt”, participants reported experiencing mixed emotions (Kawakami et al., 2013).

There is behavioral evidence to suggest that participants do indeed experience, as well as perceive, everyday emotions in response to music. Physiological and behavioral differences were found in participants listening to sad music vs. happy music, including decreased skin conductance, higher finger temperature, decreased zygomatic activity, and more self-reported sadness (Lundqvist et al., 2008). Vuoskoski and Eerola (2012) showed that sadness induced by music had similar bias effects on a word recall task and a picture judgment task as sadness induced by autobiographical recall. The results, then, are taken to mean that music can alter perception and judgment in a similar way to genuine sadness, even if listening to sad music was reported as more pleasant than recollecting a sad autobiographical memory. Neuroimaging has also provided some clarification, as sad music activated some of the regions associated with sad affective states (Mitterschiffthaler et al., 2003; Vytal and Hamann, 2010; Brattico et al., 2011). To date findings suggest that both views have merit. At times, feelings of sadness are experienced in response to sad music and can result in pleasure; at other times, sad music can bypass the associated sad feelings and directly induce a pleasurable response. Which scenario occurs most likely depends on personality, mood, and learned associations with the musical stimuli. Exploring the extent to which the emotional response to sad music overlaps with the sadness experienced in everyday life is a fertile area for further research.

### The Influence of Individual Differences, Mood, and Social Context

While sad music may be associated with various psychological rewards that are inherently pleasurable, not everyone experiences the pleasurable response all the time. In addition to the acoustic features of sad music described above, personality, mood, and the surrounding social context are all important factors in determining whether or not sad music is enjoyed. Several key personality measures are correlated with the liking of sad music, including absorption, as measured by the Tellegen Absorption Scale, and scores on subscales of the Interpersonal Reactivity Index (IRI) including fantasy and empathic concern (Garrido and Schubert, 2011). Higher scores on openness to experience and lower scores on extraversion, as defined by the Big Five Model of personality traits, were shown to be associated with the liking of sad music (Vuoskoski et al., 2011; Ladinig and Schellenberg, 2012). Trait rumination, assessed by the Rumination-Reflection Questionnaire (RRQ), was also positively correlated with enjoyment of sad music, suggesting that certain people listen to sad music not because of the resulting positive feelings, but because of some maladaptive attraction to negative stimuli (Garrido and Schubert, 2011).

Situational factors are also important. People report choosing to listen to sad music more often when they are alone, when they are in emotional distress or feeling lonely, when they are in reflective or introspective moods, or when they are in contact with nature (Taruffi and Koelsch, 2014). Some individuals report that their preference for sad music is dependent on the time of day when they listen (Taruffi and Koelsch, 2014). Other studies have shown that liking of sad music increases when the listener is repeatedly exposed to the musical excerpt while distracted or mentally fatigued (Schellenberg et al., 2008) or when the music is preceded by multiple happy-sounding excerpts (Schellenberg et al., 2012). Empirical evidence that context can have an effect on one’s emotional response to music was recently found in a study in which participants who listened to music alone showed greater skin conductance response compared to participants who listened to the same music in a group (Egermann et al., 2011).

**Figure 1:**
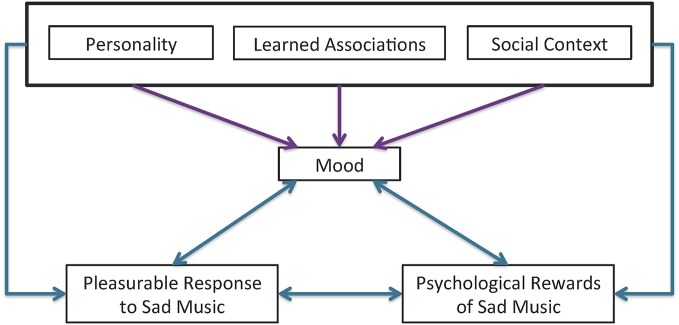
**Interaction affects between personality, social context, learned associations, and mood on pleasurable response to sad music**. Personality, learned associations, and the social context can all influence a person’s current mood (purple arrows) and the interaction of one’s mood with certain combinations of these three factors form the psychological rewards associated with sad music and, ultimately, the pleasurable response (blue arrows). The resulting pleasurable response can in turn influence current mood, as represented by the bi-directional arrow between pleasure and mood. The reciprocal nature of psychological rewards and pleasurable response is also represented by a bidirectional arrow.

Mood appears to play a role in preferences for sad music as well, though the exact nature of that role is unclear. The liking of unambiguously sad-sounding music was shown to increase after a sad-mood induction paradigm (Hunter et al., 2011). However, there is evidence to suggest that this effect may vary across individuals as some people appear to be motivated to select music that is incongruent with their current mood (i.e., selecting happy music when they are sad) while others are motivated to select music that is congruent with their mood (i.e., selecting sad music when they are sad; Taruffi and Koelsch, 2014). Whether a person selects mood-congruent or mood-incongruent music most likely depends on individual differences and social context. A previous study looking specifically at the interacting effects of mood and personality found that people who scored higher on a measure of global empathy, as well as the fantasy and personal distress subscales of the IRI, were more likely to listen to sad music when they were in a negative mood (mood-congruent). People who scored lower on measures of emotional stability were also more likely to listen to sad music when they were in a negative mood. Interestingly, global empathy scores were positively correlated with people’s preferences to listen to sad music when in a positive mood (mood-incongruent), but in this case, the perspective taking subscale, rather than personal distress, was significant (Taruffi and Koelsch, 2014). The connection between these factors and their associations with pleasurable response to sad music is summarized in Figure 1.

## The Neuroscience Perspective

Neuroimaging techniques, including functional magnetic resonance imaging (fMRI), can be used to identify areas of the brain that are activated in response to certain stimuli and thus help uncover some of the processes related to the tragedy paradox. To date, however, no study has explored the neural correlates of pleasurable sadness in response to music. In this section, we will simply draw relevant inferences from the literature.

### Sadness in the Brain

#### Perception of Sadness and Sad Mood

The neural correlates of the experience of sadness are often investigated through the use of sad-mood induction tasks. In order to induce the intended feelings, these experiments generally have the participants reflect on sad, autobiographical events and/or view stimuli that express sadness, such as sad faces or sad films (Vytal and Hamann, 2010).

Changes in mood states are associated with activity changes in the anterior cingulate cortex (ACC) and in the insular cortex, two of the main regions of cerebral cortex involved in the processing of feelings (Damasio, 1999). The two programs are interconnected (Mesulam and Mufson, 1982). Several studies using positron emission tomography (PET) or fMRI have reported heightened activity in both structures during the experience of sadness (Lane et al., 1997; Damasio et al., 2000; Lévesque et al., 2003; Habel et al., 2005). The ACC is also associated with social pain as the result of social-exclusion (Macdonald and Leary, 2005), and the processing of sad faces (Killgore and Yurgelun-Todd, 2004).

Collectively, the hippocampus, parahippocampal gyrus, and the amygdala, are presumed to be important partners in the process of emotional learning and memory. The three areas are neuroanatomically connected (Pitkänen et al., 2000) and recently several studies have shown that they are functionally connected during the processing of emotional stimuli (Hamann et al., 1999; Kilpatrick and Cahill, 2003).

The hippocampus, parahippocampal gyrus, and amygdala are also associated with unpleasant experience, as higher activity was found in these regions when participants viewed unhappy faces and thought about sad past events (Posse et al., 2003; Habel et al., 2005). Increased activity in the amygdala and parahippocampal gyrus was also found, however, during the happy mood induction task (Habel et al., 2005), suggesting that these regions are not involved in processing sadness specifically, but rather are involved in processing salient emotional stimuli (Phan et al., 2002).

Areas in the frontal lobe are also implicated in processing sadness. A recent meta-analysis found that superior frontal gyrus (BA 9), as well as an area slightly anterior to it [sometimes referred to as the medial frontal gyrus (BA 10)], were repeatedly activated during various sad mood induction tasks (Vytal and Hamann, 2010). The caudate nucleus, a region that is highly innervated by dopamine neurons and modulated by the ventral tegmental area (Faggin et al., 1990), was also involved in the same task (Vytal and Hamann, 2010). In addition, activity in the inferior frontal gyrus (IFG, BA 47) was revealed when comparing sad mood induction, directly, to happy mood induction (Habel et al., 2005).

#### Brain Correlates of Music-Evoked Sadness

The regions of the brain that are involved in processing feelings of sadness, in general, also appear to be implicated in the processing of feelings evoked by music. In a study in which participants listened to familiar music that they found sad or happy, sad pieces, compared to happy pieces, were associated with increased activation in the head of the caudate nucleus as well as the thalamus (Brattico et al., 2011). Increased activation in the thalamus has also been found during the processing of sad faces (Fusar-Poli et al., 2009).

Several studies on music and emotion have reported involvement of the hippocampus, parahippocampal gyrus, and amygdala (Blood and Zatorre, 2001; Baumgartner et al., 2006; Koelsch et al., 2006; Eldar et al., 2007). Specifically, in relation to sad music, music that induced a sad mood, judged by subjective reporting, was shown to correlate with increased blood oxygen level dependent (BOLD) signal in the hippocampus and the amygdala (Mitterschiffthaler et al., 2007).

A number of functional neuroimaging studies reported involvement of these regions in the perception of negative valence in music in particular. For example, music perceived as sad, as a result of it being either in a minor mode (Green et al., 2008) or producing low arousal and valence (Frühholz et al., 2014), was shown to correlate with increased activity in the parahippocampal gyrus. That region, along with the hippocampus, was also shown to be involved in responding to dissonant music that was found unpleasant (Blood et al., 1999; Koelsch, 2014). Because of their role in the encoding of memories, the parahippocampal gyrus, hippocampus, and amygdala may also play an important role in processing emotional events related to the music (Ford et al., 2011).

The superior frontal gyrus and the medial frontal gyrus appear to be associated with the perception of emotions in music as well; both regions were shown to be activated when contrasting the response to music in a minor key to music in a major key (Khalfa et al., 2005; Green et al., 2008).

### Aesthetic Judgments

Aesthetic judgments include both the act of deciding whether or not an auditory stimulus is aesthetic in nature, and therefore not life-threatening, as well as whether the auditory stimulus is beautiful (Jacobsen, 2006). Neuroimaging studies of aesthetic judgment generally produce activation in the frontal lobe cortices and the ACC. The orbital frontal cortex (OFC) has been shown to be involved in various decision-making processes by linking past behavior with their emotional byproducts (Bechara and Damasio, 2005). It is not surprising then, that this general area is repeatedly recruited during tasks of aesthetic judgment (Jacobsen et al., 2006; Ishizu and Zeki, 2011). Other areas of the frontal lobe, including the superior frontal gyrus, and the medial frontal gyrus (BA 9 and 10), were activated when judging the beauty of musical rhythms (Kornysheva et al., 2010) and geometric shapes (Jacobsen et al., 2006). Greater activation in the ACC is also observed when aesthetic judgments are made about both art and music (Kornysheva et al., 2010; Ishizu and Zeki, 2011).

### Pleasure in the Brain

Activation of the ventral striatum and the nucleus accumbens, during pleasurable music listening was first reported in a study by Blood and Zatorre (2001) and has since been encountered by several investigators using both fMRI (Menon and Levitin, 2005; Koelsch et al., 2006; Salimpoor et al., 2013) and PET (Brown et al., 2004; Suzuki et al., 2008). Salimpoor et al. (2011) showed that there is a direct relationship between increases in pleasure during music listening and hemodynamic activity in the right nucleus accumbens, an area that is part of the ventral striatum. The study also found that the caudate nucleus was involved in the anticipation of a pleasurable response to musical excerpts (Salimpoor et al., 2013).

In a recent fMRI study, Trost et al. (2012) found that music deemed to have positive emotional valence engages the ventral striatum selectively but in a lateralized fashion. Musical stimuli with positive valence and low arousal, those leading to tenderness, increase activity in the right ventral striatum whereas musical stimuli with positive valence and high arousal, those leading to joy, increase activity in the left ventral striatum.

Using connectivity analysis, Menon and Levitin (2005) showed significant interactions during music listening between the ventral striatum, the hypothalamus and the ventral tegmental area of the brainstem, which is involved in the production and dissemination of the neurotransmitter dopamine. The results also suggested that activation of the ventral striatum in response to pleasurable music is modulated by the activity in both the ventral tegmental area and by the hypothalamus (Menon and Levitin, 2005).

Several studies have reported activity changes in the ACC and the insula during the experience of pleasure in response to musical stimuli. In their 2001 study, Blood and Zatorre demonstrated that an increase in the subjective experience of the intensity of aesthetic chills, as well as increases in physiological measures of arousal (i.e., heart rate, muscular activity and respiration rate) occurred concurrently with a rise in cerebral blood flow within the insula and the ACC. Increased activation of the insula was also observed while participants listened to pleasant musical excerpts (Brown et al., 2004; Koelsch et al., 2006).

In an attempt identify the brain regions involved in processing specific emotions in music, Trost et al. (2012) showed that listening to classical instrumental music identified as high in arousal level and positive in valence (such as joy), led to increased respiration rate together with increased activity in the insular cortex. By contrast, listening to musical excerpts that were rated low in level of arousal, regardless of valence, correlated with increased activity in the ACC (Trost et al., 2012).

The OFC has been shown to be involved in the pleasurable response that results from music listening (Blood and Zatorre, 2001; Menon and Levitin, 2005) and the IFG was activated in response to pleasant, consonant music when compared to unpleasant, dissonant music (Koelsch et al., 2006).

In addition, there is evidence to suggest that the thalamus might be involved in the pleasurable response to emotional stimuli as increased cerebral blood flow in the region was also found to be positively correlated with intensity ratings of chills in response to pleasurable music (Blood and Zatorre, 2001) and during self-reported judgments of pleasantness across different modalities (Kühn and Gallinat, 2012).

### Summary and Neurobiological Framework

The results from the neuroimaging experiments suggest that pleasurable sadness is a consequence of several coordinated neural processes. When a sad musical stimulus reaches the brain, its emotional valence is assessed on the basis of its acoustical properties (i.e., mode, timbre, and loudness), which depends on processing in the brainstem and primary and secondary auditory cortices (Liégeois-Chauvel et al., 1998; Pallesen et al., 2005; Juslin and Västfjäll, 2008). The experience of sadness would result from previously learned associations with the auditory stimulus, the emotional content of the associated words, and the parallel changes in body state induced by the emotional process (Baumgartner, 1992; Ali and Peynircioglu, 2006; Khalfa et al., 2008; Juslin et al., 2013). Linking past experiences with emotional content recruits the network of the parahippocampal gyrus, the hippocampus and the amygdala (Killgore and Yurgelun-Todd, 2004), whereas feelings of the specific emotion, are mediated by a set of subcortical nuclei in the brain stem and basal ganglia, as well as prefrontal, anterior cingulate and insular cortices (Damasio and Carvalho, 2013).

The recognition of consonance or dissonance in the musical stimulus, previous associations and familiarity associated with the musical stimulus, and affective information, such as the emotions and feelings that are perceived or induced by the piece of music (Juslin, 2013), all serve as input for the making of aesthetic judgment, whose coordination depends on the frontal cortices, including those in the superior frontal gyrus, the middle frontal gyrus, the OFC, and the ACC (Jacobsen et al., 2006; Ishizu and Zeki, 2011).

It is often the case that judging a piece as beautiful leads to feelings of pleasure, yet this is not always true (Juslin, 2013). When a subsequent pleasurable response emerges, it can come in the form of increases in emotional arousal, which has been shown to be correlated with increased feelings of pleasure, (Salimpoor et al., 2009), and in the form of episodic memories triggered by the music which can also lead directly to pleasure (Janata, 2009). The experience of pleasure is correlated with activity in the ventral striatum, specifically in the nucleus accumbens, the caudate nucleus, and the orbitofrontal cortex (Berridge and Kringelbach, 2008).

## The Clinical Implications of Pleasurable Response to Sad Music

The most common of mood disorders, major depressive disorder (MDD), is characterized by persistent feelings of unhappiness and is often accompanied by an inability to experience pleasure (anhedonia) and a disturbed ability to describe or identify emotions (alexithymia). Investigating the response of depressed patients to negative-valance stimuli such as sad music, could provide another perspective in understanding the paradox of pleasurable sadness.

Depression appears to influence how one perceives and experiences sadness. Participants with MDD show prolonged or heightened activity in the amygdala and ACC when they process stimuli that express negative valence (Siegle et al., 2002) and increased activity in the insula and ACC when experiencing a sad mood (Mayberg et al., 1999; Keedwell et al., 2005). Given the role of these brain regions in reward processing and emotional regulation (Langenecker et al., 2007), it is possible that this pattern of activity reflects the increased intensity and salience of negative affect that is often associated with depression.

An investigation of the listening-habits of individuals diagnosed with depression produced informative results (Bodner et al., 2007; Wilhelm et al., 2013). Depressed patients expressed an intensified response to sad-sounding music when compared to healthy controls (Bodner et al., 2007). Furthermore, such patients evaluated negative-valence music as significantly more sad and angry than did healthy controls (Punkanen et al., 2011). When depressed individuals and healthy controls were asked about their reasons for listening to music, the degree to which depressed participants referenced engaging with music in order to “express, experience, or understand emotions” was significantly higher than in healthy controls (Wilhelm et al., 2013). This difference was interpreted as evidence for the notion that bringing emotions to the forefront of attention, in this case through music listening, is a way of regulating and ultimately reducing the negative affective state that is indicative of depression (Chen et al., 2007).

Neuroimaging studies have shown that depression alters the neural response to music that is found pleasurable. Significant deactivation was found in the medial OFC and the nucleus accumbens/ventral striatum when depressed patients listened to their favorite pieces of music. Of interest, no differences were found between patients and healthy controls relative to how much they reported actually enjoying the musical excerpts (Osuch et al., 2009), suggesting that the neural processing of rewarding stimuli is still effected in patients with depression even when the feelings associated with the rewarding stimuli are not. A related study found that when listening to pleasant musical stimuli, activity in the OFC, as well as the nucleus accumbens, insula, ACC, ventromedial prefrontal cortex (VMPFC), and the lateral hypothalamus, was negatively correlated with measures of anhedonia (Keller et al., 2013).

In sum, depression is associated with varied neurobiological differences in emotional processing and experience. The fact that these differences are also seen in response to music implies that experience of pleasurable sadness to aesthetic stimuli can be influenced by mental illness. Furthermore, the distinct neural activity patterns seen in depressed patients when they respond to rewarding stimuli occurred in the regions known to be involved in processing enjoyable music. This suggests that music may be well suited to target and ameliorate the diminished experience of pleasure associated with various mood disorders (Salimpoor et al., 2013).

## Discussion

### Proposed Framework

Results from various disciplines suggest that pleasure in response to sad music is related to a combination of the following concurrent factors:

1. Realization that the music stimuli have no immediate real world implications;
2. Recognition that the music stimuli have aesthetic value;
3. Experience of certain psychological benefits, which depend on the following factors, individually or in combination:
  a. Evocation of memories related to particular musical pieces or pieces similar to them;
  b. Personality traits;
  c. Social context;
  d. Current mood;

We propose that the ways in which these various factors interact to produce pleasure when listening to sad music can be understood in the perspective of homeostatic regulation. Homeostasis refers to the process of maintaining internal conditions within a range that promotes optimal functioning, well-being and survival (Habibi and Damasio, 2014). Emotions, which refer to a set of physiological responses to certain external stimuli, were selected in evolution because they favor the reestablishment of homeostatic equilibrium (Damasio and Carvalho, 2013). Feelings are experiences of the ongoing physiological state and range in their valences, from positive and pleasurable to negative and potentially painful. The valence of the feelings as well as their intensity help signify whether the associated stimulus or behavior is adaptive and should be avoided or sought in the future. Feelings are a critical interface in the regulation of life because they compel the individual organism to respond accordingly. Feelings of pleasure are a reward for achieving homeostatic balance and encourage the organism, under certain conditions, to seek out the behaviors and stimuli that produced them. Feelings of pain, in general, and mental pain specifically, on the other hand, signify homeostatic imbalance and discourage the endorsement of the associated stimuli and behaviors.

When and how music induces a pleasurable response may depend on whether a homeostatic imbalance is present at the outset and whether music can successfully correct the imbalance. There is already evidence to suggest that music has deeply rooted connections to survival (Huron, 2001). Making music encourages group cohesion and social bonding, which can lead to the successful propagation of the clan (Brown, 2000). It may also be a sign of evolutionary and sexual fitness, thus fostering mate selection (Hauser and McDermott, 2003). The fact that music listening has the capacity to communicate, regulate, and enhance emotions further suggests that music can be an effective tool in returning an organism or a group to a state of homeostatic equilibrium (Zatorre and Salimpoor, 2013).

The pleasurable responses caused by listening to sad music is a possible indication that engaging with such music has been previously capable of helping restore homeostatic balance. Given that various psychological and emotional rewards (e.g., emotional expression, emotional resolution, catharsis) are shown to be associated to a higher degree with sad music than happy music (Taruffi and Koelsch, 2014), it may be that sad music, in particular, is preferentially suited for regulating homeostasis both in general physiological terms and mental terms. This notion is further supported by the fact that listening to sad music engages the same network of structures in the brain (i.e., the OFC, the nucleus accumbens, insula, and cingulate) that are known to be involved in processing other stimuli with homeostatic value, such as those associated with food, sex, and attachment (Zatorre, 2005). This is not to say that these regions are unique to the processing of sad music or that other types of music may not be useful for homeostatic regulation. We believe that pleasurable responses to negative-valence music stimuli are best understood through their ability to promote homeostasis.

The lack of a pleasurable response to sad music might mean that either no homeostatic imbalance was present or that the musical stimuli failed to correct the imbalance. It is known that pleasure to higher order stimuli (e.g., money and music) requires learning (Berridge and Kringelbach, 2008) and thus sad music may not evoke a pleasurable response if such a stimulus never became associated, through repeated exposure, with the psychological benefits that influence homeostatic regulation.

**Figure 2:**
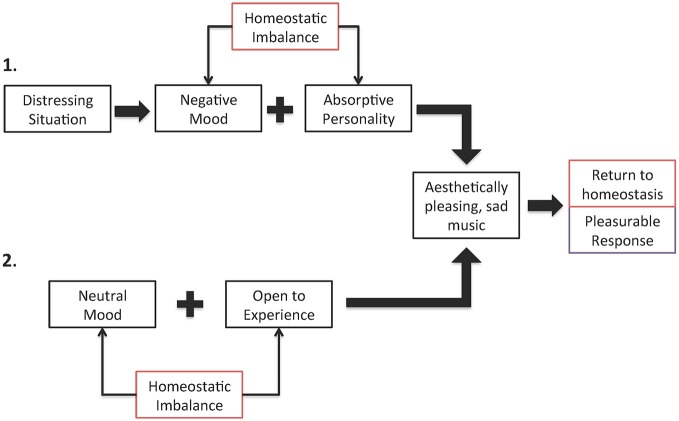
**Two examples of how homeostatic imbalance results in pleasurable response to sad music when corrected**. In example 1, a distressing situation causes a negative mood and, for a person with an absorptive personality, this is not an optimal state of being. Listening to sad music will be found pleasurable in this situation because it will allow the person to be fully engaged in an aesthetic experience, repairing their negative mood and thus resturning them to homeostasis. In example 2, for individuals who are highly open to experience, their state of optimal functioning occurs when engaged with diverse and arousing stimuli. Listening to sad music could induce a variety of emotions, serving as the desired diverse stimuli, which someone open to experience would find pleasurable because it returns them to this optimal state.

There are many ways in which a homeostatic imbalance can arise and there are numerous ways in which sad music can correct such imbalances. For example, an individual who is currently experiencing emotional distress and has an absorptive personality will be able to listen to sad music to disengage from the distressing situation and focus instead on the beauty of the music. Listening to sad music would correct the imbalance caused by emotional distress and the experience would be pleasurable. In the absence of emotional distress and the ensuing negative mood, however, a person who is highly open to experience, and prefers novel and varied stimulation, could find such diverse stimulation in sad music because of the range and variety of feelings associated with it and thus experience an optimal state of well-being (see Figure 2 for details).

Viewing the tragedy paradox in terms of humanity’s deeply rooted biological need to maintain a variety of basic psychological and physiological balances and relative stability over time, should allow researchers to focus less on the individual and situational factors associated with enjoying sad music and more on how these factors interact with each other. We believe that this comprehensive focus will ultimately permit a better understanding of the questions that persist on this issue.

### Future Directions: A. Neuroimaging Research

The published neuroimaging studies on pleasurable sadness in music are complex and difficult to synthesize due to differences in methodologies, stimuli, analysis, and participant population. While there is some agreement regarding which brain regions are involved in the process, the exact role that each region plays remains unclear. Neuroimaging studies should attempt to elucidate the contribution that different parts of the brain may make to the pleasurable response induced by music by exploring three lines of research: (1) directly comparing music that is perceived as sad but not found pleasurable with music that is perceived as sad and found pleasurable; (2) exploring how the emotional response to sad music compares to the emotional response to other types of sadness, such as sadness due to the loss of a loved one or being ostracized; and (3) considering specifically how the interaction between mood and personality alters preference for sad music.

### Future Directions: B. Using Sad Music in Music Therapy

Because of its proven ability to affect a host of neural processes, including emotions, mood, memory, and attention, music is uniquely suited to serve as a therapeutic tool for psychological intervention. The concept of using music to heal has been around for centuries, but it was only in the second half of the 20^th^ century that music therapy was first considered an established health profession with standardized academic and clinical training requirements and a board-certification program (American Music Therapy Association, 2015).^1^ Today, music therapy is used to treat a wide range of mental and physical ailments, including acute and chronic pain (Cepeda et al., 2013), brain trauma (Bradt et al., 2010), autism spectrum disorder (Gold et al., 2006), dementia (Vink et al., 2004), schizophrenia (Mössler et al., 2011), and mood and anxiety disorders (Koelsch et al., 2006; Maratos et al., 2008). Controlled clinical trials have found that music therapy, in conjunction with standard medical care, can have a significant positive effect on various symptoms associated with these illnesses (Gold et al., 2009).

Music can be particularly useful for the treatment of depression given its ability to effectively regulate mood. In general, music therapy techniques that are currently in practice for depression intervention fall into two broad categories: active therapy, which involves playing, writing, and/or improvising music, and receptive therapy, which involves passively listening to music. In active music therapy, the patient and the therapist generally create music together and then engage in a reflective discussion regarding the meaning behind the compositional experience (Erkkilä et al., 2011). In receptive music therapy, pre-selected music often serves to change the patient’s mood or to facilitate guided imagery, relaxation, or motivational exercises. In other forms of receptive music therapy, music is used to stimulate a therapeutic discussion regarding the thoughts, feelings, and memories that the music evokes (Grocke et al., 2007). Both active and receptive music therapy can be beneficial because they allow for various themes and emotions to be experienced and expressed indirectly and without the need for language (Erkkilä et al., 2011).

As previously stated, sad music, to a higher degree than other types of music, is associated with certain psychological rewards, such as regulating or purging negative emotions, retrieving memories of important past events, and inducing feelings of connectedness and comfort (Taruffi and Koelsch, 2014). Therefore, incorporating sad pieces that are found to be pleasurable into receptive music therapy could augment the efficacy of such treatments in ameliorating the symptoms of depression. Actively exploring, with the guidance of the therapist, the natural and spontaneous reactions to sad pieces of music in particular could help patients better comprehend and manage their response to negative stimuli in general, providing them with new ways of coping with sadness and connecting with others. Research into the ways in which sad music becomes enjoyable may inform existing music therapy practices for mood disorders by furthering the understanding of such disorders, offering possible mechanisms of change, and providing support for the use of personalized medicine in mental health care.

## Conclusion

The literature on the enjoyment of sad music is limited and at times conflicting, but allows us to make some general conclusions. Overall, scholars from various disciplines agree that music that conveys sadness can be found pleasurable because in art, the immediate social and physical circumstances usually associated with the negative valence, are not present. In addition, it may be that music that pertains to grief and sorrow is more often found beautiful than music that pertains to joy and happiness because it deals with eudemonic concerns such as self-expression, social connectedness, and existential meaning. Finally, sad music can help individuals cope with negative emotions in certain situations, depending on their personality, their mood, and their previous experiences with the music.

We do not yet have a detailed account of how these factors interact to produce a pleasurable response. Neuroimaging studies suggest that the response is the product of a coordinated effort between various regions of the brain known to be involved in emotional recognition, conscious feeling, aesthetic judgment, and reward processing. Future studies, in particular those that use neuroimaging techniques, should aim at manipulating mood and personality independently to determine the effect that each has on affective responses to sad music. Findings from such studies could provide new evidence for the ways in which everyday stimuli can become rewards and pave the way for new treatments of mood disorders.

## Conflict of Interest Statement

The authors declare that the research was conducted in the absence of any commercial or financial relationships that could be construed as a potential conflict of interest.

## Footnotes

^1^http://www.musictherapy.org/about/history/
